# On the Diagnosis of Diseases of the Lungs and Heart by Means of Physical Signs, &c.

**Published:** 1837-01

**Authors:** 


### ART. XIII.

1. *Zur Diagnostik der Lungen und Herzkrankheiten mittelst physicalischer Zeichen: mit besonderer Berücksichtigung der Auscultation und Percussion.* Von Dr. P. J. PHILLIPP, pract. Aerzte in Berlin.—*Berlin*, 1836.

*On the Diagnosis of Diseases of the Lungs and Heart by Means of Physical Signs; more especially those derived from Auscultation and Percussion.* By Dr. P. J. PHILLIPP, Physician in Berlin.—*Berlin*, 1836. 8vo. pp. 358.

2. *On the Diagnosis of Diseases of the Chest; based upon the Comparison of their Physical and General Signs.* By W. W. GERHARD, M.D., Physician to the Blockley Hospital; Lecturer in the Philadelphia Medical Association, &c.—*Philadelphia*, 1836. 8vo. pp. 183.

3. *A Bedside Manual of Physical Diagnosis, applied to Diseases of the Lungs, Pleuræ, Heart, Vessels, Abdominal Viscera, and Uterus.* By CHARLES COWAN, M.D. P. and E. &c.—*London*, 1836. 18mo. pp. 58.

4. *Anviisning til at kjende Lunge, og Hierte, Sygdomme ved Percussion og middelbar Auscultation.* Ved Dr. Med. S. TRIER, &c.—*Kjöbenhavn*. 1830.

*A Guide to the Diagnosis of Diseases of the Lungs and Heart, by means of Percussion and Mediate Auscultation.* By S. TRIER, M.D.—*Copenhagen*, 1830. 8vo. pp. 121.

IT will not be expected by our readers that we should, on the present occasion, enter upon the general subject of the physical diagnosis of diseases of the chest, having so recently had occasion to do so in our notice of M. Raciborski's Manual. We are, however, anxious to call the attention of our readers to the works here enumerated, not merely on account of their individual merits, but because they prove, in a very satisfactory manner, the progressive advance throughout all the nations of the earth of the knowledge of the greatest single improvement which has ever enriched practical medicine. It is creditable to this country that the first translation of the immortal work of Laennec was into English, and that, next to France, England has always taken the lead as well in the practice as in the study and furtherance of the knowledge of Auscultation. This is proved by the numerous elementary works on the subject published in this country, which, until the last year, were the only good Manuals for the student to be found in any language: and, indeed, after all the works that have recently appeared, including those at the head of this notice, we are disposed to regard Dr. Williams's *Rational Exposition*, as remodelled in the edition of last year, as still the best practical introduction to the knowledge and practice of auscultation. It is somewhat singular, notwithstanding the early publication of Laennec's discoveries in Germany, that the physicians in that country seem to have been among the last to admit their importance, or to apply them to practice; and, although the literary activity of the nation has furnished the medical profession with two translations of the *Auscultation Mediate*,—one, of the first edition, in 1822, and another of the remodelled work in 1832,—and also translations of the Treatise of Piorry and of Collin's Manual, it appears from Dr. Phillipp's Treatise that the actual practice of the new methods of diagnosis is still neglected in an extraordinary manner. "Our private practitioners (says Dr. Phillipp,) almost blush to apply the ear to the chest: in doing so, they make as great preparation as if they were going to perform a bloody operation, and, by their awkward proceeding, excite in the patient a distrust of the value of the method of exploration. In the hospitals and clinics, the physician contents himself with a hurried examination amid the surrounding bustle, paying no further attention to the results thus obtained, or rather obtaining no results to be attended to." (*Pref.* p. vi.)

We are happy, however, to state that Dr. Phillipp's work is one well calculated to alter this state of things; as it is written in the spirit best calculated to find favour in the eyes of his countrymen, being full of matter to overflowing, elaborate in its arrangement, minute in its details, and withal not without a spice of the intellectual enthusiasm which the Germans prize so highly. Indeed, we regard the treatise as excellent in every respect; and, were it not that we already possess the first-rate work of Dr. Williams, already alluded to, and have had so recently introduced into our literature the Manual of Raciborski, and the work by Dr. Gerhard, which we shall immediately notice, we would recommend the translation of Dr. Phillipp's work to some of our young friends. It is in every respect superior to Raciborski's volume, which, we are sorry to say, has lost rather than gained by being put into an English dress.

We had marked many passages in Dr. P.'s work, which had struck us as either novel or as placing the subject in a particularly clear point of view, but want of room obliges us to pass them over. We give the following statement of the relative advantages and disadvantages of mediate percussion on the *fingers* and on the *pleximeter,* as an example of the author's manner, and as containing a pretty full statement of all that can be said on both sides.

"The advocates for percussion on the finger adduce the following arguments against the employment of the artificial pleximeter:—1. The sound of the pleximeter itself modifies, in a certain degree, the normal sound of the parts to be percussed; an objection which does not apply to the finger, which consists of substances analogous to those on which it rests. 2. It is difficult, particularly in thin subjects, to adjust the pleximeter to the intercostal spaces, while the finger can be fitted to the hollows with the greatest facility. 3. The level surface of the pleximeter finds few corresponding points on the surface of the chest on which to rest; whereas the flexibility and contexture of the finger fit it to every variety of surface. 4. Percussion on the finger causes less pain; and, when the parts are very tender, we may strike on the finger with the flat hand, which is impracticable with the pleximeter.* 5. Percussion on the finger requires less dexterity than that on the pleximeter, burdens the practitioner with no instruments, and inspires the patient with no fear.

"The advocates of the artificial pleximeter maintain—1. That the more homogeneous the medium of percussion the better, and that the instrument exceeds the finger greatly in this respect. 2. That, the more solid the pleximeter, the purer are the sounds elicited from it, and the more in harmony with the subjacent parts; while the soft surface of the finger sins directly against this condition. 3. That, the thinner the pleximeter, the louder and more distinct is the sound deduced from it; but that the finger is not only too thick, but varying in its thickness in different parts. 4. That, the more level the surface to be percussed, so much easier is it to strike perpen-

* We, however, find the production of the sensation of pain enumerated afterwards by Dr. Phillipp as an important sign in some diseases, as in pericarditis and inflammation of the spinal marrow. (P. 23.)

dicularly; that the finger, being convex, is liable to afford deceptive results, particularly if one is not careful to select the centre of the phalanx. 5. That, the wider the surface of the instrument, the impulse is more divided, and is therefore less felt on individual parts: consequently, the superior extent of the pleximeter gives it great advantage in this respect. 6. That the finger is not nearly so easily fixed in its plaçe as the pleximeter.

"But, independently of all these reasons for and against the two methods, daily experience teaches us that while, on the one hand, the pleximeter can never be supplanted by the finger, persons accustomed to the use of the latter will with difficulty bring themselves to employ the former." (P. 28, 29.)

We may add, that although, in our opinion, both are requisite in particular cases, the finger possesses so many obvious advantages, that, as a general means of exploration, it will always obtain the preference.

The work of Dr. GERHARD is on the same plan as that of Dr. Phillipp, but of much less extent. It is also a very excellent Manual, and we can confidently recommend it to the English reader, as containing a concise, but on the whole correct, exposition of the whole subject of the physical diagnosis of pectoral diseases. Like Dr. Phillipp, the author has obtained his knowledge in the great Parisian school,—the only school, indeed, where such knowledge can be readily obtained; and, like him, by the publication of his acquired knowledge, he has conferred a great boon upon the literature of his country. Dr. Gerhard, like his German contemporary, is also evidently an observer of nature for himself; and, if our limits permitted, we could notice several passages in his work which are either original or which represent the subjects in a point of view more worthy of their importance than has been done by other writers. A few of these particulars we will, for the sake of our less experienced readers, briefly advert to.

We have always been anxious to inculcate attention to the physical signs most easily recognized, well knowing how much the difficulty of acquisition bars the way to knowledge of any kind. On this principle, we have constantly advised the student to commence his explorations by the study of the signs afforded by inspection, and to give the preference to percussion over auscultation. For the same reason, we consider the following observations of Dr. Gerhard as important:

"Behind the clavicles, in subjects who are neither very thin nor very corpulent, there are obvious depressions, the internal edge of them being formed by the sterno-mastoid muscle. These depressions are necessarily a little more evident in thin than in corpulent persons, and are sometimes extreme in those who are reduced to the last degree of emaciation. In very fat subjects there may be a prominence instead of a depression, without corresponding disease of the lungs. Unless the post-clavicular depressions be different on the two sides of the thorax, it is therefore very probable that they indicate no morbid state of the lungs. If one of the hollows be much deeper than the other, we infer that the corresponding portion of the lung has become contracted, and occupies less space than in the natural state; this depression is produced by adhesions between the two surfaces of the pleura, following inflammation of that membrane. As inflammation of the pleura at the summit of the lungs is almost invariably produced by a deposit of tuberculous matter in the pulmonary tissue, an important sign is thus derived from the increase of a post-clavicular depression. If the lungs be distended from dilatation of the vesicles, or if there be an effusion of liquid into the pleura, the post-clavicular hollow on the side affected is nearly effaced, or is sometimes converted into a prominence. In patients of moderate corpulency, there a depressions below the clavicles, corresponding to those above them; they are increased or diminished under the same circumstances as the post-clavicular spaces, but, from the unyielding nature of the bony walls of the chest, the difference between the two sides cannot be so great. The chest becomes gradually more prominent from the clavicles to the sixth or seventh ribs; the swell is gradual, and is partly caused by the pectoral muscles. It is a little greater on the right than the left side, except just over the region of the heart, where there is frequently a very slight local prominence. The prominence is increased by emphysema of the lungs, or by effusions of liquid into the thorax. The increased prominence is most frequently observed along the margin of the lung, which is the usual seat of emphysema. When the pericardium is distended by liquid, there is a very evident pyriform projection in the region of the heart. If the pleura be the seat of the effusion, the dilatation is more extensive and more equable. A slighter degree of prominence usually accompanies enlargement of the heart." (P. 14.)

Dr. Gerhard in general prefers the finger to the pleximeter, one of the best kinds of which he states to be a piece of common India rubber, about a quarter of an inch thick, and tolerably firm. We differ from Dr. G. when he states it as a general rule that "percussion should be made in such a manner as to produce the greatest possible sound, without giving pain." On the contrary, we are convinced that it often gives the most satisfactory results when made so slight as to be barely perceptible to the ear: in this case, it will often happen that a part which sounds pretty well under a strong impulse returns no sound, while the healthy parts yield a very distinct sound. The following directions are judicious:

"When the forefinger of the left hand is applied to the chest, the percussion may be made upon its palmar or dorsal surface. The sound is clearer, but accompanied by a slight clacking noise when the dorsal surface is percussed; it is purer, more completely hollow, and not less loud when the palmar face is struck. The latter surface is necessarily percussed when the depressions above the clavicles are examined: in that case the convex surface of the finger is readily placed in contact with the concavity of the chest. When the anterior surface of the chest is percussed, it is almost indifferent which surface of the finger is struck. When the back is examined, it is most convenient to strike upon the convex surface of the finger. Whether the pleximeter be the index-finger or a piece of caoutchouc, the fingers should strike against it in the same manner. The four fingers of the right hand should be brought into a line, and the blow be given as quickly as possible with the ends of the fingers, and not with the bulbs. The motion should be performed in the wrist and in the metacarpal joint of the fingers, the arm being perfectly passive, and much care should be taken to withdraw the fingers immediately after they have touched the pleximeter. It is frequently very convenient to use the middle finger only in percussing, if the patient be thin. The tap is then extremely light, but gives a very perfect sound." (P. 21.)

"In young children percussion affords very important signs, which are the more valuable from the comparatively little aid we can receive from the auscultation of such patients. It is best performed by tapping lightly with a single finger along the back. Very little result can be obtained from percussing them on the anterior part of the chest, which is narrow and covered by a layer of fat. Besides, almost all the diseases of the lungs in young children begin at the posterior margin or base. If the child be an infant, it should be laid upon its face, or supported, so that it may lean forwards and make the skin of the back tense." (P. 24.)

We cannot pass over, without a strong expression of dissent, Dr. Gerhard's assertion that auscultation with the naked ear has many advantages over the stethoscope; an opinion, however, in which he is supported by Dr. Phillipp, (p. 39.) In some cases it certainly has advantages; but we are positive that, as a general rule, the reverse is the truth. Almost the sole advantage of the immediate auscultation seems to be that stated by Dr. Phillipp, viz. "the quickness and facility with which the ear, when once applied, can be moved over the surface, so as at once to save time and comprehend the whole space where sounds are heard, in a single minute ausculting twenty different points." (P. 42.)

The fact mentioned in the last of the two following paragraphs is new to us: we introduce the first merely as necessary to make the second understood.

"When the ear is applied to the chest of a person in good health, a faint rushing sound is heard during the act of inspiration. When this sound is carefully analysed, it will be found to consist of two elements, more or less blended together. The first element, or the blowing sound, is that produced by the air passing through the bronchial tubes. It resembles the sound made in the mouth and fauces, when the air is quickly inhaled. It is heard most distinctly at the root of the lungs, over the trachea, and near the clavicles, especially the right. The second sound is the soft murmur caused by the expansion of the vesicles; it is the best characteristic of a healthy pulmonary tissue. This sound is termed the vesicular murmur, or the vesicular respiration, from its anatomical seat. It is best heard where the tissue of the lungs contains the greatest number of vesicles, and the smallest bronchial tubes; that is, at the base of the lungs, in the axilla, and at their anterior margin." (P. 30.)

"The singular fact that the respiration was always a little blowing at the upper part of the right lung, and not at the left, has been familiar to me for some years. I have recently made a number of dissections of the lungs, to ascertain whether this fact could be explained by their anatomical structure. It will be seen that the bronchi distributed to the upper lobe of the right lung pass nearly in a straight line from the trachea; those proceeding to the left lung make a much longer circuit, in consequence of the curve made by the left bronchium, as it passes beneath the aorta. The length of the principal bronchium, on the left side, is two inches and a half; that of the right is less than an inch and a half, measured from the middle of the bifurcation. The caliber of the bronchia passing to the right lobe is nearly double that of those on the left. There are, therefore, three circumstances which render the respiration more blowing on the right side: 1. Proximity of its bronchial tubes to the trachea. 2. Their straight course. 3. Their greater size." (P. 32.)

Chapter V. "Of the Cough and the Expectoration," is particularly good; but we remark that the author omits to notice a circumstance which we frequently meet with, and which is calculated to mislead the common observer,—viz. the apparent absence of all expectoration in phthisis, on account of the sputa being constantly swallowed by the patient. Chapter X. on Gangrene of the Lungs, and Chapter XI. on Phthisis, are also excellent, and contain as concise and accurate an account of the diagnosis as is anywhere to be found. In the case of phthisis, the author makes practical use of the difference in the normal sounds produced in the two sides of the chest, in consequence of the differing ramification of the bronchi already mentioned.

"Percussion must be made above, upon, and immediately below each clavicle; in these places a very slight deviation from the natural standard may be detected. It should, however, be recollected that a very small difference in favour of the left side does not indicate induration of the right lung, as the summit of the left is naturally a little more sonorous." * * *

"The sound in the right lung of a phthisical patient should not be regarded as morbid, unless the respiration is decidedly blowing; a slight difference, perceptible by an accurate auscultator, does not necessarily indicate disease. But, if the respiration be more blowing at the summit of the left than the right side, there can be no reasonable doubt that the lung contains tuberculous matter. The same peculiarity of structure which gives rise to a rude respiration on the right side produces a greater resonance of the voice. In the normal state the difference is extremely slight; but, if tubercles be deposited at the summit of both lungs, there will be heard a decided resonance of the voice at a much earlier period on the right than the left side." (P. 105, 106.)

The little work of Dr. Cowan is composed on a different plan, and with much humbler pretensions. The following extract from the preface clearly points out the object the author had in view in composing it; and we are satisfied, from a perusal of its contents, that it is excellently well calculated to fulfil the ends for which it is designed. We recommend it to all young auscultators; and to all, whether young or old, who have the misfortune to own a bad memory for little facts and trivial details.

"The intention of the little volume now presented to the reader is not to rival or replace any of the more extended and didactic treatises upon Auscultation or Percussion, but to furnish him with a summary of the best established signs for the treatment of disease, supposing a previous acquaintance with their individual value and interpretation, as well as with the nature and application of the means best adapted for their detection." * * *

"We feel convinced that the number of students who extensively benefit by the use of the means to which we are referring is very limited, compared with those who possess but a theoretical familiarity with their details; while there is an intermediate class, who evince a practical acquaintance with the subject to a certain extent, but who relinquish their confidence in physical investigation when opposed to the complications which the protean progress of disease so frequently engenders. It is to the last two classes that this little Manual is principally addressed, with the hope of marshalling to their aid, in the moment of practical need, those physical indications which are immediately suited to their wants; and, by fixing the attention upon the more prominent and frequent characteristics of particular diseases, to assist in relieving the mind from the anxious embarrassment which seldom fails to accompany a less definite and visionary knowledge." (*Pref.* p. i. ii.)

We have placed the little work of Dr. Trier on our list, merely as enabling us to record Denmark among the foreign countries which have added to their national literature the history of the discoveries of Auenbrug, Laennec, and Piorry. This small treatise has been published six years, and, although it contains a faithful digest of the principal facts, it is on too limited a scale to be a sufficient guide for the practitioner. It was produced under the direction of the celebrated Professor Bang, and is founded on the works of Laennec, Piorry, Andral, and Collin. It is a performance highly creditable to the author, and has, we have reason to believe, been very useful to his countrymen.

---

